# IMT504 protects beta cells against apoptosis and maintains beta cell identity, without modifying proliferation

**DOI:** 10.14814/phy2.15790

**Published:** 2023-08-11

**Authors:** Ayelén Converti, María Silvia Bianchi, Mario D. Martinez, Alejandro D. Montaner, Victoria Lux‐Lantos, María Marta Bonaventura

**Affiliations:** ^1^ Instituto de Biología y Medicina Experimental (IBYME‐CONICET) Buenos Aires Argentina; ^2^ CONICET‐Universidad de Buenos Aires, UMYMFOR Buenos Aires Argentina; ^3^ Departamento de Química Orgánica, Facultad de Ciencias Exactas y Naturales Universidad de Buenos Aires Buenos Aires Argentina; ^4^ Instituto de Ciencia y Tecnología (ICT‐Milstein) Buenos Aires Argentina; ^5^ Universidad Nacional de San Martin (UNSAM), ECyT Buenos Aires Argentina

**Keywords:** apoptosis, oligodeoxynucleotides, proliferation, transcription factors, β‐Cells

## Abstract

We have demonstrated that oligodeoxynucleotide IMT504 promotes significant improvement in the diabetic condition in diverse animal models. Based on these results, here we evaluated whether these effects observed in vivo could be due to direct effects on β‐cells. We demonstrate by immunofluorescence that IMT504 enters the cell and locates in cytoplasm where it induces GSK‐3β phosphorylation that inactivates this kinase. As GSK‐3β tags Pdx1 for proteasomal degradation, by inactivating GSK‐3β, IMT504 induces an increase in Pdx1 protein levels, demonstrated by Western blotting. Concomitantly, an increase in *Ins2* and *Pdx1* gene transcription was observed, with no significant increase in insulin content or secretion. Enhanced Pdx1 is promising since it is a key transcription factor for insulin synthesis and is also described as an essential factor for the maintenance β‐cell phenotype and function. Dose‐dependent inhibition of H_2_O_2_‐induced apoptosis determined by ELISA as well as decreased expression of *Bax* was also observed. These results were confirmed in another β‐cell line, beta‐TC‐6 cells, in which a cytokine mix induced apoptosis that was reversed by IMT504. In addition, an inhibitor of IMT504 entrance into cells abrogated the effect IMT504. Based on these results we conclude that the β‐cell recovery observed in vivo may include direct effects of IMT504 on β‐cells, by maintaining their identity/phenotype and protecting them from oxidative stress and cytokine‐induced apoptosis. Thus, this work positions IMT504 as a promising option in the framework of the search of new therapies for type I diabetes treatment.

## Graphical abstract

Oligodeoxynucleotide IMT504 enters pancreas beta cells through the thiol‐mediated uptake and locates in the cytoplasm where it phosphorylates GSK‐3β inactivating it. This leads to increased expression of PDX1 protein and mRNA, increased Ins1 expression, and protection from cytokine‐and peroxide‐induced apoptosis.

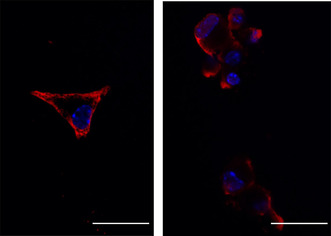

## 1 INTRODUCTION

Type I diabetes mellitus (T1D) is defined by the autoimmune destruction of pancreatic β‐cell mass, and its management is centered on insulin replacement, maintaining tissue sensitivity to insulin and glucose homeostasis. The pathophysiology of type 2 diabetes mellitus (T2D) is a combination of insulin resistance and, at later stages of the disease, reduction in β‐cell mass. At the time of T2D diagnosis, diabetic patients seem to have already lost about 50% of their pancreatic beta cells (Marrif & Al‐Sunousi, 2016). Considering this background information, the current challenge for diabetes research is to achieve increases and/or maintenance of β‐cell mass.

Adult β‐cells are mostly quiescent; the limited regenerative capacity of β‐cells has led many researchers to search for alternative approaches to replenish lost or dysfunctional β‐cells. Immunomodulatory oligodeoxynucleotides (ODNs) are synthetic molecules that activate immune cells. Two classes of ODNs have been identified, CpG (Krug et al., 2001) and non‐CpG ODNs. Our group has identified a class of non‐CpG ODN, being IMT504 the prototype of this class (Bianchi et al., 2016). IMT504 promoted marked improvement in rat models of bone injury (Hernando et al., 2007), neuropathic pain (Coronel et al., 2008), and sepsis (Chahin et al., 2015). Regarding diabetes, we have previously demonstrated that IMT504 promotes significant improvement in the diabetic condition in various animal models: For example, induction of β‐cell recovery, inhibition of leukocyte infiltration into the islet, improvement on glucose and insulin blood levels, decrease in apoptosis, and insulitis and infiltration of CD45+ leukocytes into the pancreas (Bianchi et al., 2010, 2012, 2016, 2021). TLR‐9 is the receptor that recognizes DNA oligonucleotides from the CpG ODN class. Unpublished results from our group demonstrated that while the effects of a CpG ODN were completely abrogated when transfecting cells with a TLR‐9 siRNA, IMT504 effects were not, suggesting it does not utilize this pathway to enter cells. Very recently, Laurent et al. (2021)) demonstrated that oligonucleotides with a phosphorothioate backbone, as is the case with IMT504, enter mammalian cells (e.g., HeLa Kyoto cells) much more efficiently than nonmodified DNA and that they utilize a thiol‐mediated uptake. Whether this mechanism is responsible for IMT504 incorporation into β‐cells is still unknown and will be addressed in this work.

Of interest, IMT504 meets the three criteria for a useful drug in autoimmune diabetes, as proposed by Giannoukakis et al. (2008): (1) it significantly maintains/restores β‐cells, (2) it markedly inhibits insulitis, and (3) it is easy and cheap to synthesize and safe to administer (Franco et al., 2014; Hernando‐Insua et al., 2010).

Important physiological functions have been attributed to Pdx1 in mature β‐cells, including glucose sensing, insulin biosynthesis, and insulin exocytosis (Johnson et al., 2003). Also Pdx1 has been reported to modulate the expression of several β‐cell genes including those coding for insulin (Chakrabarti et al., 2002; Ohlsson et al., 1993). The developmental role of Pdx1 persists into adulthood through its lifelong action on the maintenance of islet mass, architecture, and plasticity, processes that involve the interaction of β‐cell neogenesis, differentiation and apoptosis (Chakrabarti et al., 2002). Pdx1 decrease predisposes islets to apoptosis, suggesting an important role of this factor on β‐cell wellness (Ohlsson et al., 1993).

Pancreatic β‐cells are known to be more susceptible to damage from oxidative stress compared with other tissues due to the low expression of antioxidant enzymes. Under oxidative stress, β‐cells lose their ability to synthesize insulin and enter an apoptotic stage. In addition, β‐cells are also susceptible to cytokine‐induced apoptosis in an inflammatory environment as occurs in T1D (Hubber et al., 2021). In T1D and T2D, β‐cell mass is decreased and the main mechanism underlying this is increased β‐cell apoptosis (Butler et al., 2003; Thomas et al., 2009). Apoptosis pathways consist of the extrinsic, or death receptor, pathway and the intrinsic, or mitochondrial, pathway. The mitochondrial pathway regulates the balance between the antiapoptotic and proapoptotic Bcl‐2 family of proteins (Tomita, 2016).

While increased apoptosis is the major defect leading to a decrease in β‐cell mass in T2D, β‐cell replication and new islet formation seem to be conserved (Butler et al., 2003); this is probably also true for T1D. Thus, therapeutic approaches designed to regulate apoptosis could be a significant new development in the management of diabetes. Bianchi et al. demonstrated that in diabetic animals the immunomodulatory ODN IMT504 induced a decrease in β‐cell apoptosis (Bianchi et al., 2016, 2021).

In the present study, we investigated whether IMT504 had direct effects on β‐cells by studying its incorporation into the cells, its effects on gene expression, intracellular signaling pathways, proliferation, and apoptosis on the MIN6B1 cell line.

## 2 MATERIALS AND METHODS

### 2.1 Oligodeoxynucleotide

HPLC‐purified IMT504 was purchased from Biosynthesis (Texas). This ODN that has phosphorothioate internucleotide linkages was suspended in depyrogenated water, assayed for lipopolysaccharide contamination, and kept at −20°C until used. The IMT504 sequence is 5´ TCATCATTTTGTCATTTTGTCATT 3′ (Hernando et al., 2007).

### 2.2 Cell lines and culture

The cell line derived from mouse β‐cells, MIN6B1, was kindly provided by Professor Philippe Halban (Lilla et al., 2003). MIN6B1 were cultured in Dulbecco's Modified Eagle's Medium (DMEM, Gibco) containing 20 mM glucose, supplemented with 15% fetal bovine serum (FBS), 71 μM β‐mercaptoethanol and streptomycin/penicillin (DMEM basal medium), at 37°C in a humidified atmosphere of 95% air and 5% CO_2_. Cells were harvested using trypsin/EDTA solution (GIBCO cat#: 25200–072). Cells were used from passages 18–25. For all experiments the number of independent cultures was *n* = 5–8.

A second β‐cell line (β‐TC‐6) was used to corroborate the results obtained with MIN6B1 cells. The mouse pancreatic β‐cells line, Beta‐TC‐6 (ATCC CRL‐11506), was purchased from the American Type Culture Collection (ATCC) and cultured in (DMEM, Gibco), containing 20 mM glucose, sodium bicarbonate (1.5 g/L) supplemented with 15% FBS and streptomycin/penicillin, at 37°C in a humidified atmosphere of 95% air and 5% CO_2_. Cells were used from passages 8–12. For the experiment, the number of independent cultures was *n* = 4.

### 2.3 Cytochemical localization of IMT504 in MIN6B1 cells by immunofluorescence (IF)

Cells were seeded onto a glass coverslip covered with polylysine (50,000 cells/well). Thereafter cells were incubated with 0.01 μg/μl of IMT504 N‐terminal linked Texas Red (Bio‐Synthesis Inc.) for 5, 30, 60 min, and 4 h and fixed in 4% paraformaldehyde. Confocal microscopy was performed to localize cells staining for IMT504 (red) and DAPI (nuclear staining, blue, VECTOR laboratories). Confocal images were obtained by an IX83 Olympus microscope.

### 2.4 Immunoblotting

Western Blot (WB) analysis for pGSK‐3β, GSK‐3β, pAkt, Akt, PCNA, Pdx1, and Actin were performed in MIN6B1 cells. Cells were seeded in a 6‐well plate (500,000 cells/well) and incubated with IMT504: 2 (IMT2), 4 (IMT4), and 8 μg/mL (IMT8) for 24 and 48 h, or 4 μg/mL (IMT4) for 5, 15, 30, and 60 min. Protein lysates (50 μg) were separated by SDS‐PAGE gels and transferred onto nitrocellulose membranes. Membranes were blocked with 0.5% bovine serum albumin (BSA) and incubated overnight at 4°C with primary antibodies [anti‐pGSK‐3β (Santa Cruz Biotechnology Inc., CA, sc‐373,800, 1:100, this is a mouse monoclonal antibody epitope corresponding to a short amino acid sequence containing Ser 9 phosphorylated GSK‐3β. Phosphorylation in Ser is known to cause inactivation of this enzyme), anti‐pAkt (Cell Signaling, #9271, 1:1000), anti‐PCNA (Santa Cruz Biotechnology, Inc., CA, sc‐25,280, 1:1000), and anti‐Pdx1 (Developmental Studies Hybridoma Bank)], followed by 2 h, room temperature incubation with HRP‐linked secondary antibodies [antimouse (Vector Laboratories, CA, 1:4000), anti‐rat (Santa Cruz Biotechnology, Inc., CA, 1:4000), or anti‐rabbit (Vector Laboratories, CA, 1:4000)]. Membranes were stripped and reused for total GSK‐3β, total Akt, or Actin determination: membranes were incubated overnight with primary antibodies [anti‐GSK‐3β (Santa Cruz Biotechnology Inc., CA, sc‐7291, 1:200), anti‐Akt (Santa Cruz Biotechnology Inc., CA, sc‐1618, 1:500), and anti‐Actin (Calbiochem, Merck, CP01, 1:5000)] followed by 1 h incubation with HRP conjugated antimouse (Vector Laboratories, CA, 1:4000) or anti‐goat (Santa Cruz Biotechnology, Inc., CA, 1:4000).

Specific proteins were visualized by the enhanced chemiluminescence method using WB detection reagent followed by image analysis with G‐Box documentation system (Syngene, Unitek, BA). Images were integrated using Image‐J and expressed relative to the nonphosphorylated or actin protein.

### 2.5 Gene expression analysis

Cells were seeded in 6‐well plates (500,000 cells/well) and incubated with different concentrations of IMT504: IMT2, IMT4, or IMT8 or DMEM Basal Medium as control (C) for 12 h, 24 h, and 48 h. When evaluating the expression of apoptotic genes, cells were incubated with the same concentrations of IMT504 along with H_2_O_2_ 0.75 μM (H_2_O_2_) for 24 h. Total RNA was extracted using TriReagent (Molecular Research Center, USA) according to the manufacturer's instructions. 2 μg of RNA was reverse‐transcribed in a 20‐μl reaction using MMLV reverse transcriptase (Ref. 28025–013, Invitrogen, CA, USA) and oligo(dT) primers (Ref. B071‐40, Bio‐dynamics, Buenos Aires, Argentina) and quantified using the Nanodrop spectrophotometer (Thermo Scientific NanoDrop 2000).

For quantitative real‐time PCR (qPCR), primer sets were chosen for the specific amplification of the murine genes listed in Table 1.

**TABLE 1:** Genes and primer sets: *Ins1* and *Ins2*, preproinsulin 1 and 2, respectively.

Gene	RefSeq No.	Forward primer (5′‐3′)	Reverse primer (3′‐5′)
*Ins1*	NM_008386	AAGCTGGTGGGCATCCAGTAACC	GTTTGGGCTCCCAGAGGGCAAG
*Ins2*	NM_001185083	TACACACCCATGTCCCGCCGT	TTCTGCTGGGCCACCTCCAGT
*Pdx1*	NM_008814	GCTCACGCGTGGAAAGGCCAGGA	CTCTCGGTCAGGTTCAACATCACTGCCAGCT
*MafA*	NM_194350	GAGGAGGTCATCCGACTGAAA	GCACTTCTCGCTCTCCAGAAT
*Isl1*	NM_021459	GCGCTCATGAAGGAGCAACTA	TGATGCTGCGTTTCTTGTCC
*NeuroD1*	NM_010894	ACAGACGCTCTGCAAAGGTTTG	GCGGATGGTTCGTGTTTGAAAG
*Bcl2*	NM_009741.5	TTCTTTGAGTTCGGTGGGGTC	GGGGCCATATAGTTCCACAAA
*Bax*	NM_007527.3	CTAGCAAACTGGTGCTCAAGGC	AGTGTCCAGCCCATGATGGTTC
*Ppib*	NM_011149	GACCCTCCGTGGCCAACGAT	ACGACTCGTCCTACAGATTCATCTC

Abbreviations: Bax, BCL2‐associated X protein; *Bcl2*, B cell leukemia/lymphoma 2; *Isl1*, ISL1 transcription factor, LIM/homeodomain; *MafA*, v‐Maf musculoaponeurotic fibrosarcoma oncogene family, protein A; *NeuroD1*, neurogenic differentiation 1; *Pdx1*, pancreatic and duodenal homeobox 1; *Ppib*, cyclophilin B.

Target cDNA quantification was performed in a total volume of 10 μL using 5 X HOT FIREPolEvaGreenqPCR Mix Plus (Solis BioDyne, Tartu, Estonia) according to the manufacturer's protocol. Amplification was carried out in a CFX96 Touch Real‐Time PCR Detection System (Bio‐Rad, CA). Each sample was analyzed in duplicate.

### 2.6 Cell proliferation

Cells were seeded onto a 96‐well plate (10,000 cells/well) and incubated for 24 or 48 h with or without stimuli IMT2, IMT4, and IMT8. All stimuli were evaluated in quadruplicates. After the selected time periods MTS reagent was added following the manufacturer's instructions. Proliferation was measured with the Cell Proliferation Kit I (MTS, Cat#: 11465007001‐Roche‐SIGMA‐ALDRICH). Development of color was measured in a spectrophotometer at 492 nm. Results were expressed as Arbitrary Units (A.U.: Absorbance 405‐Absorbance 492).

### 2.7 Synthesis of inorganic polysulfides (IPS)

Inorganic polysulfides (IPS) have been demonstrated to inhibit the uptake of oligonucleotides with a phosphorothioate backbone, as is the case with IMT504, by mammalian cells (Cheng et al., 2020; Laurent et al., 2021). The IPS was synthesized following a previously reported procedure with some modifications (Cheng et al., 2020). Brieflly, elemental sulfur (150 mg, 0.6 mmol) was added to a solution of sodium sulfide (160 mg, 2.0 mmol) in Milli‐Q water (10.0 mL) and the reaction mixture was stirred for 1 h at 70°C under argon atmosphere. The corresponding orange solution was cooled down to room temperature and centrifuged (10 min at 6000 rpm) to remove any solid. Finally, the supernatant was directly used as stock solution of inorganic polysulfides (0.2 M, Na_2_S_n_, *n* = 2–8).

### 2.8 Cell apoptosis

MIN6B1 cells were seeded in 96‐well plates (7500 cells/well) and incubated with H_2_O_2_ 0.75 μM (Xiang et al., 2016) with or without IMT2, IMT4, and IMT8 for 24 h. Apoptosis levels were evaluated using the Cell Death Detection ELISA^PLUS^ (Sigma Cat. No 11774425001), according to the manufacturer's protocol. Development of color was measured in a spectrophotometer at 405 and 492 nm. Results were expressed as Arbitrary Units (A.U.: Absorbance 405 nm‐Absorbance 492 nm).

For immunocytochemical (ICC) determination of apoptosis, MIN6B1 cells were cultured as described above, with the IMT4 dose only. TUNEL labeling was performed using the DeadEndFluorometric TUNEL System (#G3250, Promega) according to the manufacturer's instructions. Confocal images were obtained by an IX83 Olympus microscope.

β‐TC‐6 cells were seeded in 96‐well plates (20,000 cells/well) and exposed to IFN‐α, IL‐1β, IL‐6, and TNF‐α (5 ng/mL per cytokine) with or without IMT4 or IMT8, with or without 25 μM IPS for 24 h. IPS alone was also used as a control. Cytokines were purchased from R&D Systems: rhIL‐6 (Cat. # 206‐IL/CF), rhIL‐1β (Cat. # 201‐LB/CF), rhIFN‐γ (Cat. # 285‐IF) and TNF‐α (Cat. # 210‐TA). All treatments contained FITC Annexin V Dye (1 μL/well, BioLegend cat # 640945) and apoptosis levels were evaluated using the Incucyte® Live Cell Analysis System (Sartorius). Results are expressed as the percent increase in each well of apoptosis after 24 h with regard to basal apoptosis levels (0 h).

### 2.9 Insulin determination

Cells were seeded in 24‐well plates (200,000 cells/well) in DMEM basal medium. After 48 h, cells were incubated with or without stimuli IMT2, IMT4 for 48 h. Insulin content in culture media/cells was determined by RIA with the usual lab protocol (Anti‐pig insulin, developed in Guinea pig, SIGMA, cat # I 8510, 1:60000) (Bonaventura et al., 2008).

### 2.10 Statistical analysis

All data are expressed as means ± SEM. Statistical analysis was performed with Statistica '99 Edition. The differences between means were analyzed by the unpaired Student's *t*‐test or by two‐way ANOVA, followed by Newman–Keuls test or Tukey HSD test for unequal N. For multiple determinations within the same cells, ANOVA with repeated measures design was used. To analyze differences between percentages of more than two groups, data were arcsine transformed and analyzed by ANOVA. *p* < 0.05 was considered statistically significant.

## 3 RESULTS

### 3.1 Localization of IMT504 in MIN6B1 cells

**FIGURE 1:**
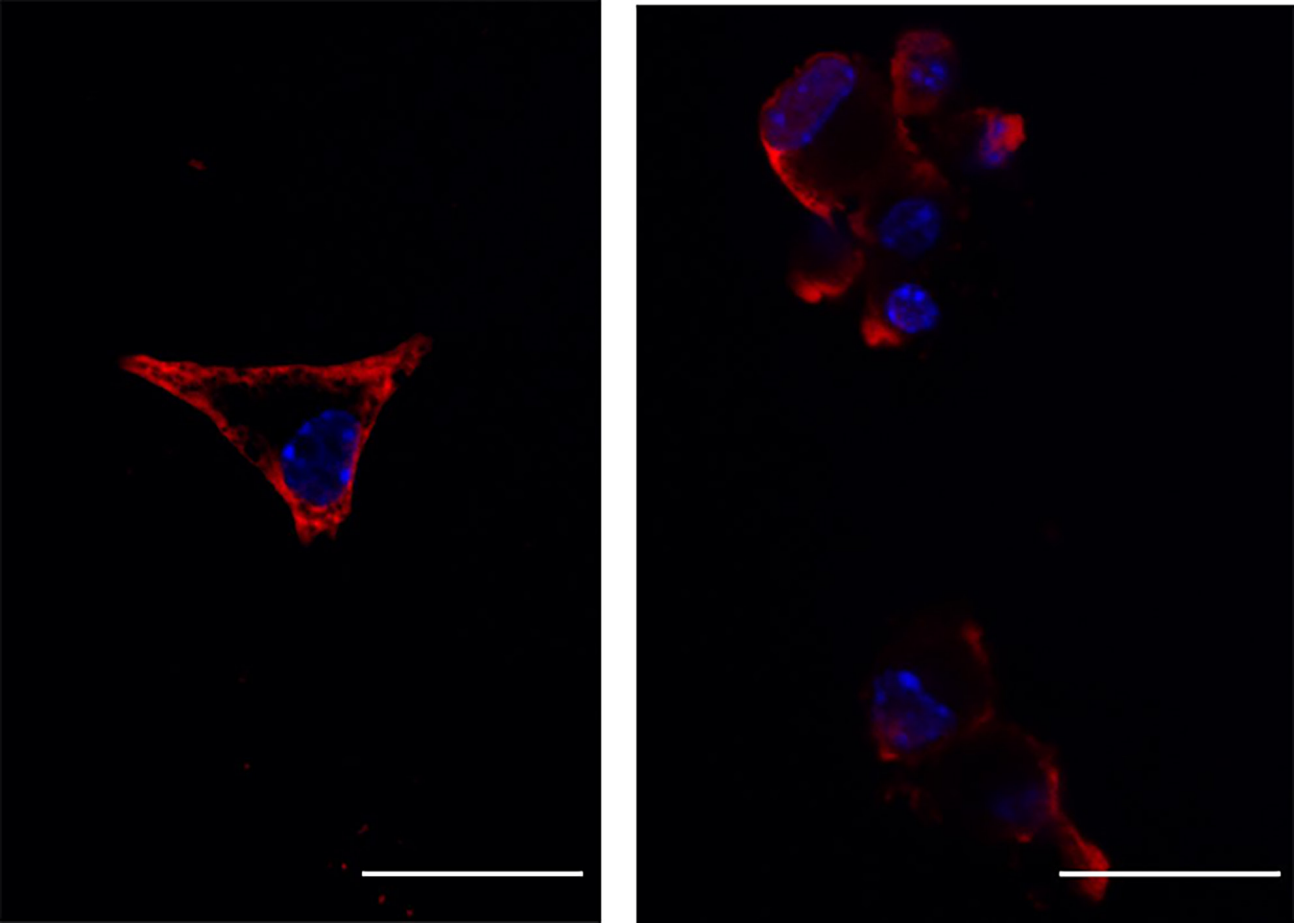
Confocal microscopic analysis of two examples of IMT504‐Texas Red incorporation into MIN6B1 cells after 4 h incubation; nuclei stained with DAPI (blue), 60X, bar = 20 μM.

The main aim of this work was to elucidate whether IMT504 had a direct effect on β‐cells. Therefore, we first investigated whether IMT504 interacted in any way with β‐cells. Fluorescent cytochemistry was successfully used to demonstrate the localization of IMT504 in the pancreatic β‐cells. β‐cells were already strongly positive for oligodeoxynucleotide IMT504 at early time points (5 min) after the initiation of the incubation with IMT504 conjugated to the Texas Red fluorophore (Texas Red‐IMT504). 4 h after the addition of Texas Red‐IMT504, the label was still predominantly cytoplasmic. Figure 1 shows a representative image with nuclei stained in blue (DAPI) and IMT504 in red. Oligonucleotide IMT504 was found both, associated to the cytoplasmic membrane and in the cytoplasm. However, it was mostly undetectable inside the nucleus. It remains unclear whether IMT504 was located exclusively in the cytosol or also in organelles.

### 3.2 IMT504 regulates Pdx1 gene and protein expression levels in MIN6B1 cells: Effects on insulin expression

**FIGURE 2:**
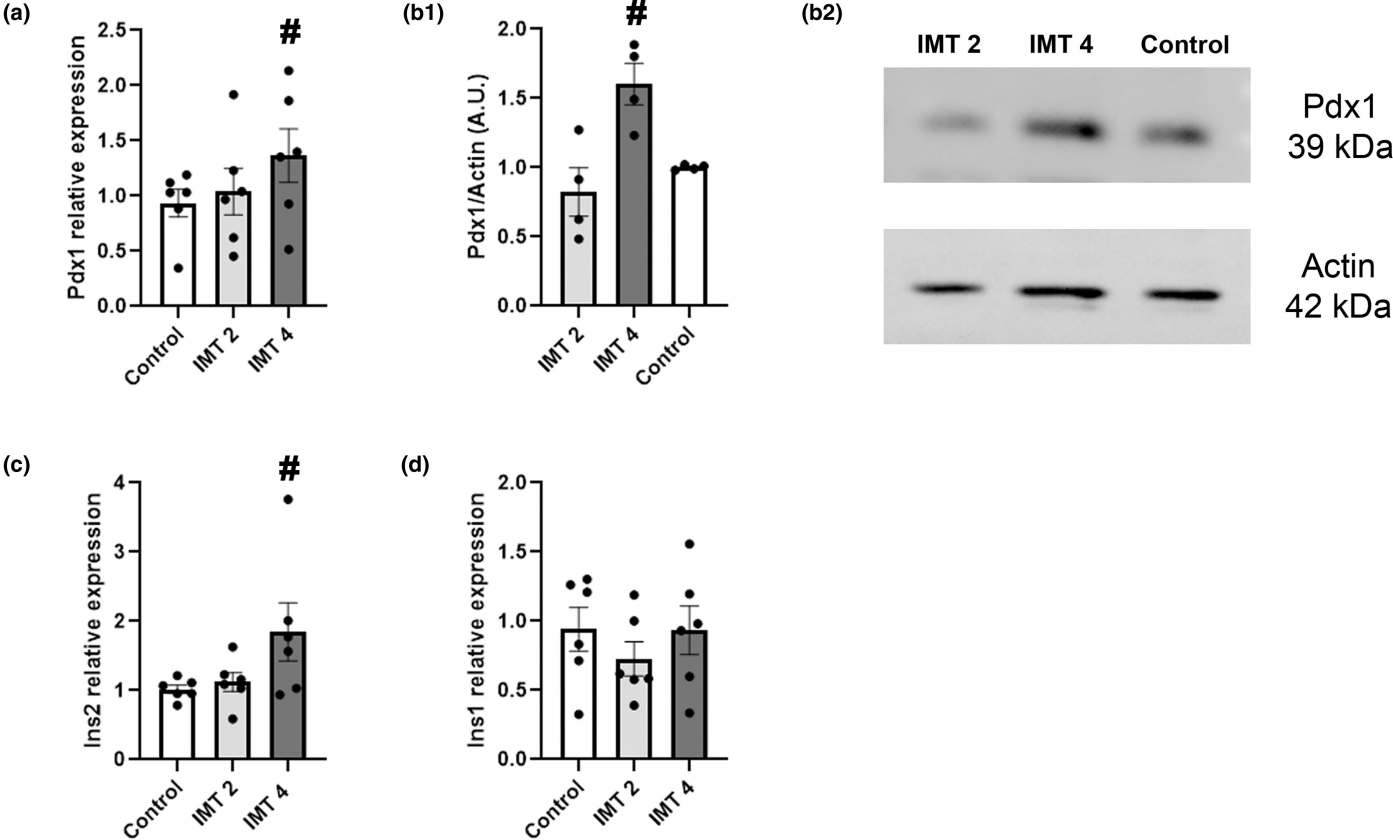
(a). qPCR Gene expression evaluated in MIN6B1 cells treated with different concentrations of IMT504 for 48 h. *Pdx1/Ppib*: ANOVA with repeated measures, *p* < 0.02, #: IMT4 different form C, *p* < 0.05, *n* = 6. (b) Western blot analysis of Pdx1 protein expression in MIN6B1 with IMT2 and IMT4 for 48 h. B1: Pdx1/Actin ratio was significantly increased with IMT4. ANOVA with repeated measures, *p* < 0.03, #: IMT4 different from C and IMT2, *p* < 0.05, *n* = 4; B2: Representative blot of Pdx1 and Actin expression. (c,d). qPCR Gene expression evaluated in MIN6B1 cells with different concentrations of IMT504 for 48 h. (c) *Ins2/Ppib* increased in cells incubated with IMT4; ANOVA with repeated measures, *p* < 0.05, #: IMT4 different from C and IMT2 *p* < 0.05 *n* = 6. (d) *Ins1/Ppib* did not vary among the experimental groups; ANOVA with repeated measures, NS, *n* = 6.

In Figure 2 we show the expression of the most important transcription factor (TF) involved in *Ins* transcription, Pdx1. Pdx1 is also described as a marker of mature β‐cell identity and its expression is essential for maintenance of the β‐cell phenotype. We found an increase in *Pdx1* mRNA expression (Figure 2a) after 48 h incubation with IMT4. We also evaluated *NeuroD1*, *MafA*, and *Isl1* expressions, finding no statistical differences between groups (data not shown). We next evaluated whether the increase in *Pdx1* expression was translated into an increase in Pdx1 protein. Pdx1 protein levels were markedly increased after 48 h of stimulation with IMT4 (Figure 2b) in agreement with *Pdx1* gene expression.

As we found an increase in *Pdx1* gene and protein expression levels, we next evaluated the expression of genes encoding for insulin. We observed that IMT504 increased the expression of *Ins2* after 48 h of incubation with IMT4 (Figure 2c). No differences were observed in *Ins1* expression, but this gene is not as predominant as *Ins2* in mice (Babaya et al., 2006) (Figure 2d). Although transcription of *Ins2* is markedly increased after 48 h of stimulation with IMT4, there was no statistical difference on insulin cell content or insulin secretion among the control cells and those treated with IMT2 or IMT4, determined by RIA (insulin content ng/300.00 cells: ANOVA, ns; Control (C)): 6.4 ± 0.7; IMT2: 6.5 ± 0.7; IMT4: 5.6 ± 0.8. Insulin secretion/insulin content, ANOVA ns; (C: 0.19 ± 0.04; IMT2: 0.17 ± 0.04; IMT4: 0.25 ± 0.04).

Gene expression was also determined at 12 and 24 h of incubation with the ODN, but no significant differences were observed at these time points (not shown).

### 3.3 IMT504 modulates GSK3β in MIN6B1 cells

We then investigated the possible mechanism of action of IMT504 in β‐cells. IMT504 has been shown to modify GSK3β and Akt phosphorylation in other cell types (unpublished results). Since GSK3β and Akt participate in the regulation of β‐cell physiology, we evaluated these signaling pathways in MIN6B1 cells in the presence of IMT504.

**FIGURE 3:**
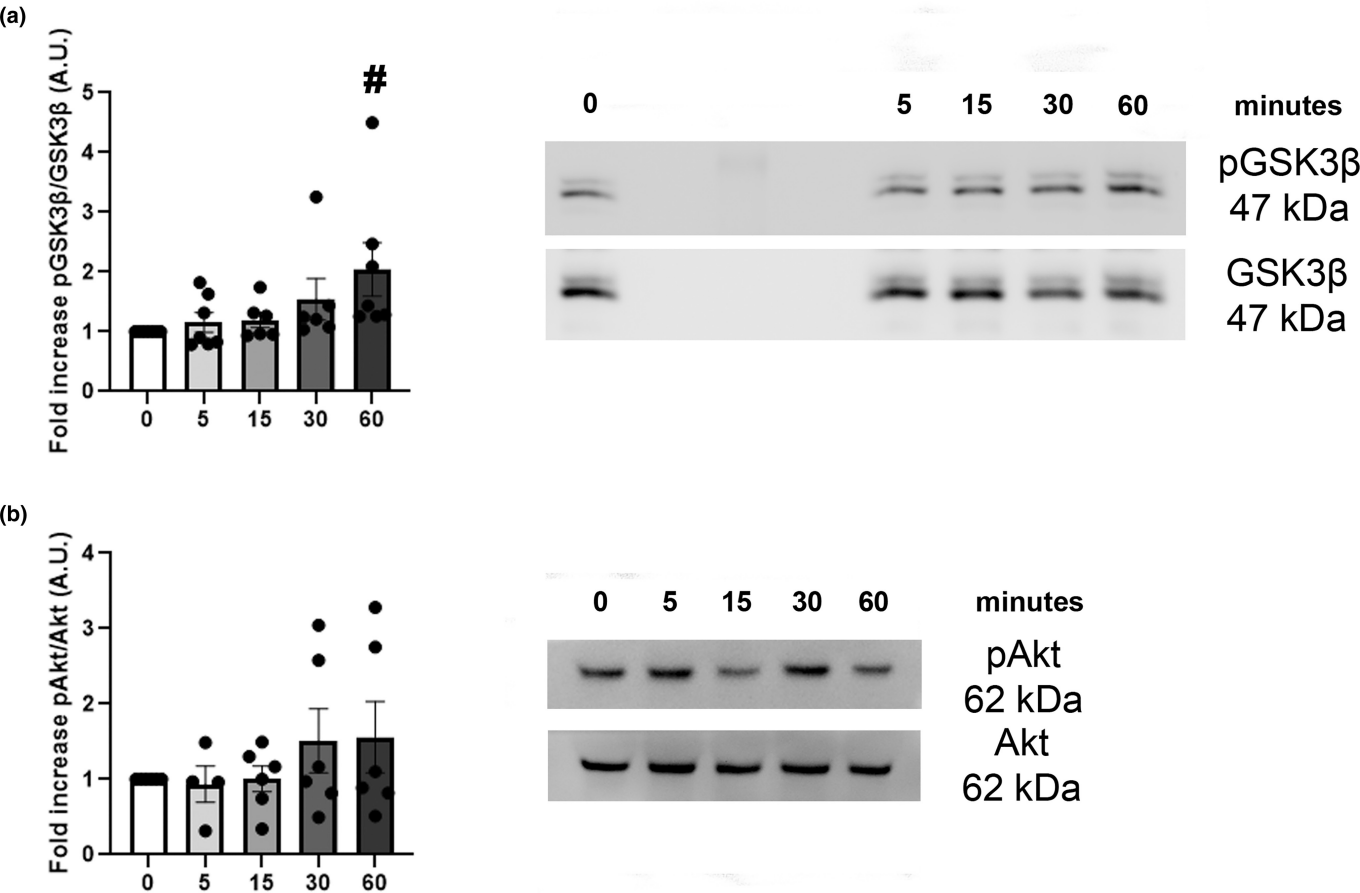
Western Blot analysis demonstrating the effects of IMT504 on GSK3β and Akt phosphorylation in MIN6B1 cells. Cells were incubated with IMT4 for a short term (5–60 min). (a) At 60 min, the IMT504‐induced pGSK3β/GSK3β ratio was significantly increased compared to the basal pGSK3β/GSK3β ratio (0 min = 1). ANOVA with repeated measures, *p* < 0.03. #: 60 min different from 0 min, *p* < 0.03, *n* = 6–7. (b) No significant differences in the pAkt/Akt ratio were observed. ANOVA with repeated measures, NS, *n* = 4–6.

We found that IMT504 induced GSK3β Ser 9 phosphorylation in MIN6B1 cells in a time‐dependent manner (Figure 3a). By contrast, IMT504 did not stimulate Akt phosphorylation in MIN6 cells after 5‐min, 15‐min, 30‐min, or 60‐min stimulation (Figure 3b). Time‐dependent phosphorylation of GSK β was maximal at 60 min. These data suggest that IMT504 could exert its actions through a pathway that includes GSK3β phosphorylation and may produce effects at the level of gene expression of different genes characteristic of β‐cells.

### 3.4 Effects of IMT504 on MIN6B1 cell proliferation

**FIGURE 4:**
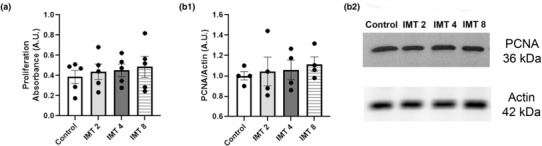
Effect of IMT504 on MIN6B1 cell proliferation (a). MTS. Incubation with IMT2, IMT4, and IMT8 for 24 h had no significant effect on MIN6B1 cell proliferation compared to C cells. ANOVA with repeated measures, NS, *n* = 5. **B1‐2**. WB analysis demonstrated no effect of IMT504 (IMT2, IMT4, and IMT8 for 24 h) on PCNA/Actin expression ratio. ANOVA with repeated measures, NS, *n* = 4.

In vivo studies in T1D diabetes models suggested that IMT504 induced active proliferation, mainly in β‐cells (Bianchi et al., 2010, 2016). Subsequently, we determined the effect of IMT504 on MIN6B1 proliferation. We stimulated MIN6B1 cells with different concentrations of IMT504 and evaluated the proliferation rate at 24 h post stimuli finding no significant differences (Figure 4a, ANOVA, NS). Proliferation was also evaluated at 48 h but IMT504 again showed no effect (not shown).

To further analyze the effects of IMT504 on proliferation, PCNA protein levels in pancreatic β‐cells were also evaluated, showing no differences between treatments, in agreement with results described above (Fig. 4B1 and 4B2, ANOVA, NS).

### 3.5 IMT504 protects beta cells against apoptosis

**FIGURE 5:**
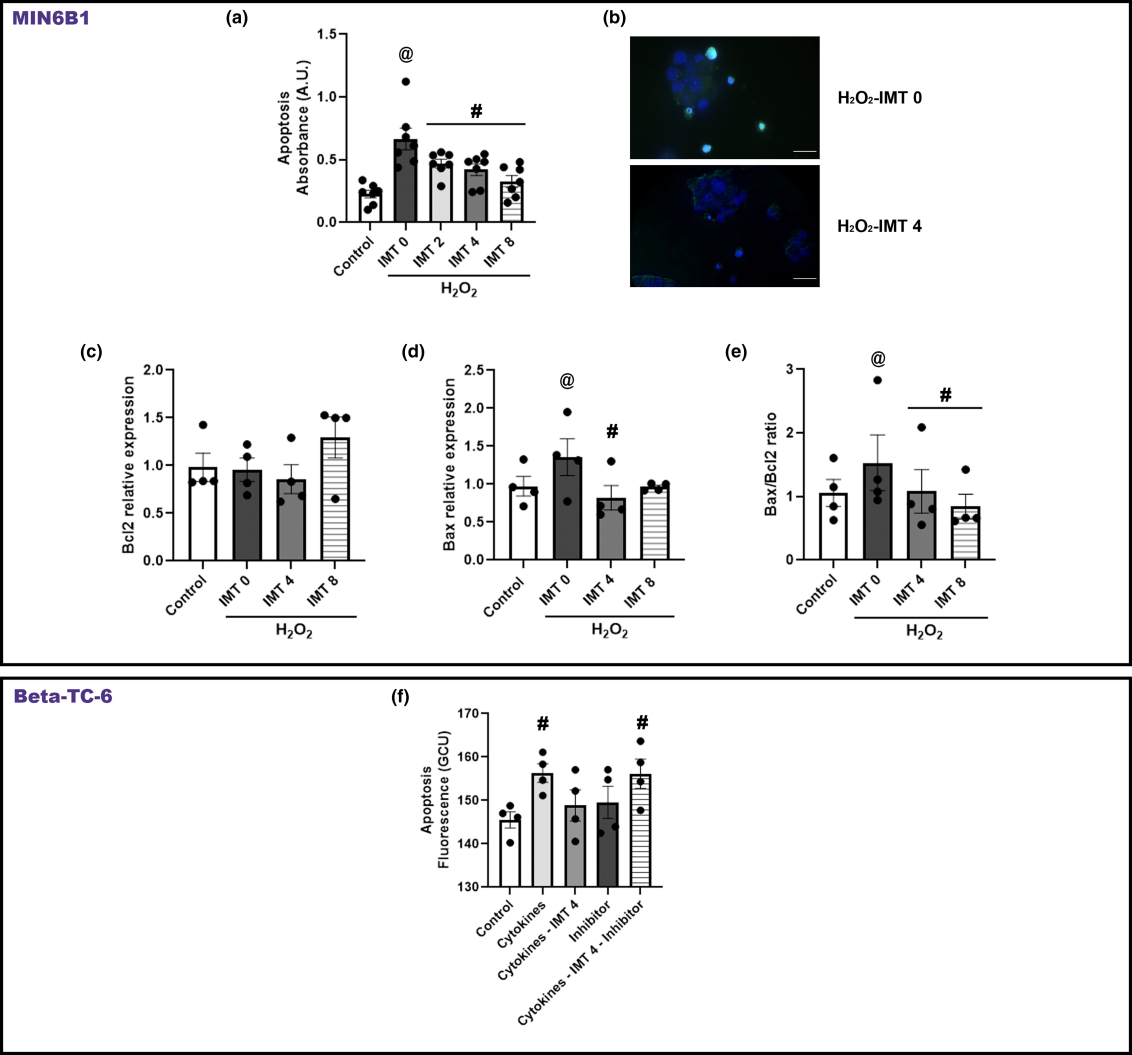
Effects of IMT504 on β‐cell Apoptosis. (a). Cell Death Detection ELISA: MIN6B1 cells were treated with 0.75 μM H_2_O_2_ for 24 h with or without IMT504. ANOVA with repeated measures, *p* < 0.01, *n* = 7, @: H_2_O_2_‐IMT0 different from C, *p* < 0.05; #: H_2_O_2_‐IMT2, H_2_O_2_‐IMT4, H_2_O_2_‐IMT8 different from H_2_O_2_‐IMT0 *p* < 0.05. (b) Representative images of TUNEL assay 40X, C with H_2_O_2_ (upper panel), IMT4 with H_2_O_2_ (lower panel). Regular nuclei (blue) and apoptotic nuclei (turquoise), bar = 20 μM. (c–e) qPCR Gene expression evaluated in MIN6B1 cells treated with different concentrations of IMT504 and a low concentration of H_2_O_2_ (0.75 μM) for 24 h. (c). *Bcl2/Ppib* did not vary among the experimental groups; ANOVA with repeated measures, NS, *n* = 4. (d) *Bax/Ppib* increased in cells incubated with H_2_O_2_ and decreased to C levels with H_2_O_2_‐IMT4; ANOVA with repeated measures, *p* < 0.05. @: H_2_O_2_‐IMT0 different from C, *p* < 0.04; *: H_2_O_2_‐IMT4 different from H_2_O_2_‐IMT0 group, *p* < 0.04, *n* = 4. (e): *Bax/Bcl2* ratio increased in cells incubated with H_2_O_2_ and decreased to C levels with H_2_O_2_‐IMT4 or H_2_O_2_‐IMT8; ANOVA with repeated measures, *p* < 0.05. @: H_2_O_2_‐IMT0 different from C, *p* < 0.05; *H_2_O_2_‐IMT4 and H_2_O_2_‐IMT8 different from H_2_O_2_‐IMT0_,_ *p* < 0.04 and 0.01, respectively, *n* = 4. (f) Beta‐TC‐6 cells treated with the cytokine mix for 24 h with or without 4 μg/mL IMT504, in the presence or absence of 25 μM IPS. Apoptosis was determined by nuclei staining with Annexin V with the Incucyte® Live Cell Analysis System. ANOVA with repeated measures, *p* < 0.02. *: Cytokine Mix (CYT) significantly different from control and CYT‐IMT054. (g): Beta‐TC‐6 cells were treated with the cytokine mix for 24 h with or without 8 μg/mL IMT504, in the presence or absence of 25 μM IPS. ANOVA with repeated measures, NS.

As described above, IMT504 improves β‐cell number in in vivo diabetes models, where it has also been shown to inhibit apoptosis (Bianchi et al., 2016). As we found no effect on cell viability/proliferation, we next evaluated the effect of IMT504 on β‐cell apoptosis. H_2_O_2_ is a well‐known trigger of apoptosis through oxidative stress involving the mitochondrial pathway (Xiang et al., 2016). Moreover, H_2_O_2_ has been shown to induce MIN6B1 cell death by apoptosis (Liu et al., 2020). Treatment with a low concentration of H_2_O_2_ (0.75 μM) was found to be sufficient to induce significant apoptosis in MIN6B1 cells (Figure 5a). Treatment with IMT504 concentration‐dependently protected the MIN6B1 cells from H_2_O_2_‐induced toxicity. In fact, H_2_O_2_‐induced cell death was nearly completely blocked by treatment with IMT8 (Figure 5a).

Supporting the above results, TUNEL assay demonstrated that IMT504 induced a significant decrease in cell apoptosis. Figure 5b shows a representative image where the IMT504 effect was evidenced by the decrease in the number of apoptotic cells (turquoise nuclei)/total cells (blue nuclei).

Apoptosis induced by mitochondrial dysfunction is regulated by Bcl‐2 proteins, comprising proapoptotic and antiapoptotic members (Choi & Woo, 2010). In an effort to better understand the effect of IMT504 on H_2_O_2_‐induced apoptosis, we evaluated the expression of apoptosis regulator genes *Bcl2* and *Bax*. *Bcl2*, an antiapoptotic marker, was unaltered between groups (Figure 5c, ANOVA, NS). By contrast, H_2_O_2_ upregulated the expression of proapoptotic marker, *Bax*, while pretreatment with IMT504 attenuated this effect in MIN6B1 cells (Figure 5d, *p* = 0.019). Likewise, the *Bax*/*Bcl2* ratio was also significantly decreased with IMT504 stimulation regarding the H_2_O_2_–induced increase (Figure 5e).

To further determine, on the one hand, whether the antiapoptotic effects observed with IMT504 were specific of MIN6B1 cells or more general of β‐cells and, on the other hand, to determine whether another β‐cell proapoptotic stimulus typical of T1D, such as a cytokine mix, could also be reversed by IMT504, Beta‐TC‐6 cells were incubated for 24 h with a cytokine mix (TNFα, IL‐1β, IL‐6, and INFγ) in the presence or absence of IMT504 (4 or 8 μg/mL) and Annexin V to determine the apoptotic nuclei. Moreover, to determine whether thiol‐mediated uptake of the oligodeoxynucleotide IMT504 was involved in the effects observed, cells were also incubated the 25 μM IPS either alone or in combination with cytokines plus IMT504. The results of this experiment show that in Beta‐TC‐6 cells a cytokine mix induced significant apoptosis that was totally reversed by coincubation with 4 μg/mL of IMT504. Including IPS in the incubation mixture, abolished the antiapoptotic effect of IMT504, demonstrating that it enters the cells via the thiol‐mediated uptake; IPS alone showed no effect on apoptosis (Figure 5f). When cells were incubated with the cytokine mix and 8 μg/mL of IMT504 a similar pattern was observed though the analysis did not attain statistical significance due to increased variation between experiments. Although lacking statistical significance, it seems that this IPS concentration was unable to block the effect of 8 μg/mL IMT504 (Figure 5g). Interestingly, 1 μM H_2_O_2_ did not induce apoptosis in Beta‐TC‐6 cells (not shown).

## 4 DISCUSSION

T1D is characterized by a selective and progressive autoimmune destruction of pancreatic β‐cells, leading to insulin deficiency and resultant hyperglycemia. People with T1D depend on lifelong exogenous insulin administration. Nevertheless, not even current insulin pharmacological formulations can fully prevent the complications that account for the morbidity and mortality of diabetic patients. Restoration of endogenous glycemic control in these patients will likely require a safe therapy for use in humans that confers immunoregulation and β‐cell benefits by replacing or maintaining the functional integrity of the natural insulin‐producing cell. It is therefore of vital importance for public health and of high economic interest for the pharmaceutical industry to develop drugs capable of reducing the autoimmune attack while maintaining or recovering the functionality of the patient's own beta cells.

With this in mind, and since IMT504 has both immunomodulatory and regenerative properties, our group started to evaluate this ODN as a possible therapy for T1D in several animal models, finding very promising results in terms of beta cell recovery, improvement on glucose and insulin blood levels, and decrease in beta cell apoptosis, and insulitis and infiltration of CD45+ leukocytes into the pancreas (Bianchi et al., 2010, 2012, 2016, 2021). Nevertheless, these in vivo experiments did not hint to a possible direct effect of IMT504 on β‐cells.

Here we studied whether IMT504 had direct effects on β‐cells in the MIN6B1 cell line. Our results indicate that IMT504 enters β‐cells, showing a marked accumulation in the cytoplasm that does not seem to translocate into the nucleus, at least during the time‐lapse evaluated. Furthermore, the uptake of the ODN takes place in a very short time, demonstrating that internalization occurs very fast. The images obtained by immunofluorescence suggest that the incorporation of IMT504 would occur mainly by mechanisms that do not include endocytosis since the main stain is cytoplasmic and not associated with vesicles, even at times as short as 5 min. As described below, IMT504 enters β‐cells by the thiol‐mediated uptake.

Several authors have described that the TF Pdx1 plays a central role in the function of mature β‐cells, as it has been described as a key regulator of insulin gene expression. Mutations of the *Pdx1* gene in humans result in defective insulin secretion, and the development of one form of maturity onset diabetes of the young 4 (MODY4) in humans (Fujimoto & Polonsky, 2009; Semache et al., 2014). Additionally, conditional deletion of *Pdx1* in 80% of mature β‐cells in an inducible conditional knockout model induces diabetes by switching the identity of β‐cells to an α‐cell‐like phenotype (Gao et al., 2014). Therefore, Pdx1 is critical for pancreas development, β‐cell maturation, and function (Jara et al., 2020). We show that both Pdx1 gene expression and protein levels were increased in MIN6B1 cells exposed to IMT504 for 48 h, indicating that IMT504 favors the maintenance of β‐cell identity. One molecular mechanism of action through which chronic hyperglycemia can cause worsening β‐cell function is via decreased protein expression of Pdx1 (Robertson, 2004), whereas the positive effects of glucose on insulin transcription may be a result of its enhancement of Pdx1 nuclear translocation (Macfarlane et al., 1999). Based on our results, we postulate that IMT504 may protect β‐cells exposed to high glucose levels during diabetes development, as suggested by our in vivo experiments.

We further evaluated whether this increase in Pdx1 correlates with an increase in insulin encoding genes. There are two genes that encode for insulin protein: *Ins1* and *Ins2*. Previous reports have postulated that the second one is the most relevant in mice (Babaya et al., 2006). We observed significantly increased expression of *Ins2* in MIN6B1 cells incubated with IMT504 for 48 h, while *Ins1* remained unchanged. Moreover, others TF involved in the regulation of *Ins2* expression, such as *Mafa*, *NeuroD1*, and *Isl1* (Bernardo et al., 2008) were not affected by IMT504 treatment. These results reinforce the crucial role of Pdx1 in beta cell functionality, that is, insulin synthesis.

Due to the results obtained with Pdx1, we then studied the possible intracellular signaling pathways that are activated upstream of Pdx1. It has been described that Gsk‐3β decreases Pdx1 expression at the protein and transcriptional levels. In this regard, phosphorylation of Ser61 and Ser66 of Pdx1 through Gsk‐3β appears to target Pdx1 for proteasomal degradation, thereby decreasing its half‐life (Fujimoto & Polonsky, 2009). Moreover, Sacco et al demonstrated in human islets and INS1 cells that glucotoxicity triggers GSK3‐dependent Pdx1 degradation and that GSK3 inhibitors restore Pdx1 expression levels (Sacco et al., 2019). In our experiments, IMT504 induced GSK3β phosphorylation in MIN6B1 cells in a time‐dependent manner. Phosphorylation of GSK3β in Ser 9 renders this enzyme inactive. So, IMT504‐induced inactivation of GSK3β by phosphorylation may have decreased the proteosomal degradation of Pdx1, leading to the observed increase in Pdx1 protein levels. Nevertheless, the inactivation of GSK3β and stabilization of Pdx1 protein levels do not explain the increase in Pdx1 mRNA expression in these cells. Several GSK3β inhibitors are currently being studied as potential drugs for diabetes treatment, and based on our results, IMT504 could be a promising candidate on that list.

Pdx1 has also been proposed to regulate β‐cell proliferation (Jara et al., 2020; Spaeth et al., 2017). Nevertheless, IMT504 did not affect proliferation in MIN6B1 cells. Given that we have previously described recovery of the β‐cell mass in diabetic animals due to the action of IMT504 and we did not see here an increase in β‐cell proliferation, we evaluated whether IMT504 had any effect on apoptosis in this cell type.

Pancreatic β‐cells are known to be more susceptible to oxidative stress compared to other tissues, since they express very low levels of antioxidant enzymes while generating high levels of ROS due to rapid glucose metabolism (Baumel‐Alterzon et al., 2021). Under this condition, which is a prominent feature of diabetes, β‐cells lose their ability to synthesize insulin and enter an apoptotic stage. Thus, pharmacological interventions targeting the inhibition of oxidative stress‐induced β‐cell apoptosis may be a key step in the prevention/amelioration of diabetes (Lu et al., 2013). Apoptosis was induced by a low concentration of H_2_O_2_ in MIN6B1 cells and was concentration‐dependently reversed by treatment with IMT504. IMT8 completely restored β‐cells to control cell levels lacking H_2_O_2_. Furthermore, chronic exposure to high levels of ROS can stimulate β cell apoptosis through the mitochondrial “intrinsic” pathway by activation of the proapoptotic members of the Bcl‐2 family (Baumel‐Alterzon et al., 2021). In response to oxidative stress, Bax permeates the mitochondrial outer‐membrane, leading to the efflux of cytochrome c to the cytoplasm, triggering apoptosis (McArthur & Kile, 2018). We observed an increase in *Bax* expression and in the *Bax*/*Bcl2* ratio upon treatment of MIN6B1 cells with H_2_O_2_, which was dose dependently reduced by IMT504 treatment. Therefore, the β‐cells recovery observed in vivo may be due to direct IMT504 protection from oxidative stress‐induced damage, in addition to other indirect effects.

To further determine, on the one hand, whether the antiapoptotic effects observed with IMT504 were specific of MIN6B1 cells or more general of β‐cells and, on the other hand, to determine whether another β‐cell proapoptotic stimulus, such as a cytokine mix, could also be reversed by IMT504, a new set of experiments was undertaken in Beta‐TC‐6 cells. While 0.75 μM H_2_O_2_ induced significant apoptosis in MIN6B1 cells, 1 μM H_2_O_2_ did not induce apoptosis in Beta‐TC‐6 cells, demonstrating differential responses between cell lines. Nevertheless, the cytokine mix induced significant apoptosis in Beta‐TC‐6 cells that was reversed by IMT504. Including the inhibitor of the thiol‐mediated uptake (Laurent et al., 2021) in the culture medium completely abolished the antiapoptotic effect of IMT504, demonstrating for the first time that this oligodeoxynucleotide with a phosphorothioate backbone uses this mechanism to enter the β‐cells. These results support the antiapoptotic effect of IMT504 in β‐cells.

In conclusion, our results demonstrate that IMT504 has an effect not only at the immunological level, as we described previously (Bianchi et al., 2016), but also directly on β‐cells in the diabetic condition. These results are in agreement with our previous results in STZ‐induced diabetic rats, in which the immune system does not play a role in diabetes development, and a marked IMT504‐induce recovery of β‐cells was observed (Bianchi et al., 2010). Thus, our data support the fact that part of the effects of IMT504 observed in vivo are due to direct effects on β‐cell function, increasing its potential as a therapy for diabetes.

## AUTHOR CONTRIBUTIONS

A.C. and M.M.B. collected and analyzed the data. M.M.B, A.D.M, M.S.B., and V.L.L. designed experiments. M.D.M synthesized the IPS to inhibit thiol‐mediated uptake. M.M.B and V.L.L. wrote the manuscript. All authors reviewed and edited the manuscript.

## CONFLICT OF INTEREST STATEMENT

The authors have nothing to disclose.

## Data Availability

The data that support the findings of this study are available from the corresponding author upon reasonable request.
